# "Fighting the system": Families caring for ventilator-dependent children and adults with complex health care needs at home

**DOI:** 10.1186/1472-6963-11-156

**Published:** 2011-07-04

**Authors:** Knut Dybwik, Terje Tollåli, Erik W Nielsen, Berit S Brinchmann

**Affiliations:** 1 Department of Anaesthesiology, Nordland Hospital, 8092 Bodø, Norway; 2 Medical Department, Nordland Hospital, 8092 Bodø, Norway; 3 Institute of Clinical Medicine, University of Tromsø, 9037 Tromsø, Norway; 4 Faculty of Professional Studies, University of Nordland, 8049 Bodø, Norway

## Abstract

**Background:**

An increasing number of individuals with complex health care needs now receive life-long and life-prolonging ventilatory support at home. Family members often take on the role of primary caregivers. The aim of this study was to explore the experiences of families giving advanced care to family members dependent on home mechanical ventilation.

**Methods:**

Using qualitative research methods, a Grounded Theory influenced approach was used to explore the families' experiences. A total of 15 family members with 11 ventilator-dependent individuals (three children and eight adults) were recruited for 10 in-depth interviews.

**Results:**

The core category, "fighting the system," became the central theme as family members were asked to describe their experiences. In addition, we identified three subcategories, "lack of competence and continuity", "being indispensable" and "worth fighting for". This study revealed no major differences in the families' experiences that were dependent on whether the ventilator-dependent individual was a child or an adult.

**Conclusions:**

These findings show that there is a large gap between family members' expectations and what the community health care services are able to provide, even when almost unlimited resources are available. A number of measures are needed to reduce the burden on these family members and to make hospital care at home possible. In the future, the gap between what the health care can potentially provide and what they can provide in real life will rapidly increase. New proposals to limit the extremely costly provision of home mechanical ventilation in Norway will trigger new ethical dilemmas that should be studied further.

## Background

Despite substantial variations between countries and variations within each country [1], an increasing number of individuals with complex health care needs now receive life-long and life-prolonging ventilatory support at home. This "hospital care at home" has implications for both the community health care services [2] and family members [3]. Families usually take on the role of primary caregivers [4] and are often greatly involved in the provision of complex care and advanced medical procedures [5]. Previous research has focused primarily on describing the families' experiences when caring for technology-dependent children, even though the majority who receive home mechanical ventilation (HMV) are adults. On one hand, these studies uncover the families' positive experiences because the treatments prolong life and increase the quality of life of their loved ones [6]. Families describe deep and enriching experiences they that do not regret [7]. The value of life is so important that family members would choose HMV again if they were faced with the same decision [8]. However, on the other hand, families face extreme emotional and social burdens [8]. Family caregivers experience exhaustion and anxiety, especially when they must be available around the clock for a tracheostomised family member dependent on continual ventilatory support.

Many problems are related to the families' encounters with the health and social care services [7], bad attitudes and a lack of competent health care professionals (HCP) [9]. When the ventilated individual is a child, the parents' roles change and they experience the double role of being both a parent and a nurse [5]. Parents may experience a lack of privacy [10,11], a certain degree of isolation in their own home [7,12], great emotional stress and overwhelming responsibility [8]. The tremendous amount of attention and care needed by the ventilator-dependent individual can have negative consequences on the relationships between spouses and siblings in the household [7].

The aim of this study was to explore the experiences of families giving continual care to family members dependent on HMV. Studies performed in a variety of geographical and health care settings can potentially uncover a wider range of experiences [7]. We studied the experiences within the Norwegian population because, to date, this has never been done and other researchers have recommended further research in this area [13]. Previous research has focused mainly on the experiences of families caring for ventilator-dependent children, but in this study we included the families of both children and adults. Insight into these experiences will be an important resource for hospital personnel establishing HMV, planning hospital discharges, and who will follow up HMV patients after hospital discharge. In addition, this knowledge can be useful for patients and families preparing for HMV. The local health care services in the patient's community can also use this information to provide better care to both the families and ventilator-dependent individuals.

## Methods

### Study design

Qualitative research methods using a Grounded Theory influenced approach and in-depth interviews were selected to explore the experiences of family members.

### Study setting

The study was carried out in various regions of Norway. Norway has a public financial health system in which specialized hospitals establish ventilation support and follow-up even after hospital discharge. Community health care services, in cooperation with family caregivers, have responsibility for the daily care provided in the patient's home. In Norway, family members can be paid employees as part of the health care team caring for the HMV-dependent family member.

### Sampling

In classic Grounded Theory, the ongoing analysis steers the sampling and the data collection (theoretical sampling), but this can prove difficult to implement in practice. Strauss and Corbin state that, "as with all research, there is the ideal way of conducting a study and the practical way (or that for which one has to settle)" [14]. At first, we interviewed seven families. At a later stage, we decided to interview a further three families to achieve theoretical saturation, meaning when we determined that new information did not add new knowledge or insight.

### Recruitment

Families were recruited via four specialized hospitals in various regions in Norway with the assistance of nurses or hospital consultants responsible for patient care. Families meeting the following criteria were included: the HMV-dependent family member had a tracheostomy, complex care needs, and required mechanical ventilation either full-time or for the majority of the day. Each family was contacted by hospital staff to provide information and obtain consent to pass on their details to the researcher. All of those contacted gave their willingness to participate and the main author then contacted them via telephone to arrange an interview at their home.

### Participants

A total of 15 family members with 11 ventilator-dependent individuals (three children and eight adults) were recruited for the study. All of the ventilator-dependent individuals had chronic neuromuscular disease (NMD), like, amyotrophic lateral sclerosis, Facioscapulohumeral muscular dystrophy, Duchenne muscular dystrophy, infantile myofibromatose, limb-girdle muscular dystrophy, nemaline myopathy, spinal muscular atrophy type 1 and syringomyelia. Ages varied from eight to 78 and although the degree of disability varied, all were capable of being mobilised in a wheelchair during the day. One of the participants was a mother to two ventilator-dependent adults.

Each of the families received care assistance from the community health care services and two of the families received care from private or commercial nursing agencies. HCP had to be present all week, around the clock. Typical daily activities of the home care provider included help with personal hygiene and meals (in some cases via gastrotomy and a feeding pump), removal of airway secretions with the use of suction and a mechanical in-exsufflator (cough machine), use of a wheelchair, medication administration, and using communication aids. Each child attended school and HCP assisted with transport. HCP had to be present at school to monitor the child's condition, help them to the restroom or remove airway secretions, if necessary. HCP also accompanied the patients to routine hospital check-ups or even vacation. Excluding one interview, when one of the patients had recently moved to an assisted-living facility, all of the participants were somehow involved in the daily care. They also performed highly technical clinical procedures, trained HCP, and organised the care. Some of the family members were on the community payroll for being a part of the health care team caring for the ventilator-dependent family member. Participant and the ventilator-dependent family member characteristics are presented in Table 1, but detailed information was excluded to ensure participant and patient confidentiality.

**Table 1:** Participants and patient characteristics.

Interview #	Participant's relationship to the ventilator-dependent family member	Years with tracheostomy and home mechanical ventilation
1	Mother and father	10

2	Mother and father	10

3	Wife	10

4	Husband	11

5	Mother	16 + 16

6	Mother and father	8

7	Mother and father	11

8	Wife	12

9	Wife and daughter	3

10	Husband	11

### Data collection

Ten in-depth interviews, varying from 60 to 90 minutes, were carried out from the period of October 2009 to April 2010. Interview guides with a few open-ended questions was used for the general direction of the interviews (see additional file 1 "Interview guide used for the first in-depth interviews"). The participants were encouraged to speak openly about the experiences that were most important, relevant, and problematic to them. To gain a deeper understanding of the experiences, and according to the principles of the Grounded methodology, we edited the discussion guide based on a continual analysis and comparison of collected data before arriving to the next family. Memos were taken during the research process or immediately after data collection. Data collection and analysis occurred simultaneously.

### Data analysis

All of the interviews were recorded and transcribed verbatim. In accordance with Grounded Theory, transcripts and memos were analysed several times and line-by-line (open coding) to find the words or phrases used by the participants to describe their experiences. In the final stage of data analysis, we manually sorted the codes into larger categories and subcategories (selective coding), and all codes were compared. This is what is called the "constant comparative method" [15,16]. A core category and three subcategories of what was most important for the participants were found. Quotes correlating to each of the categories are collected in separate tables and cross-referenced to participants and interview numbers.

### Ethical considerations

All of the potential participants received a letter containing information about the study, that participation was voluntary and that they could withdraw from the study without obligation or giving notice. At the interview, all participants gave their written consent to participate. All gathered material has been treated anonymously. Participants acquainted with the main author, KD, were interviewed by a co-author, BSB. The study was approved by the Norwegian Social Science Data Services (20781) and Regional Committee for Medical Research Ethics (2009/293-5).

## Results

The core category, "fighting the system", became the central theme as family members were asked to describe their experiences. In addition, we identified the subcategories, "lack of competence and continuity", "being indispensable" and "worth fighting for".

### Fighting the system (Core category)

All of the participants expressed a strong desire for their ventilator-dependent family members to be given the opportunity to live the most optimal and normal life as possible despite serious respiratory failure and severe disabilities. Despite considerable effort and commitment, the families were still dependent on their local community health care services to ensure their loved one received proper care. Based on the extensive experience the participants had, they were mostly focused on describing the continual struggle with the community health care services, or "the system", as they called it. Even though a member of their family was completely dependent on highly advanced medical procedures and technological equipment, the "fight against the system" seemed to be the most problematic issue.

Participants complained about the lack of involvement they had in decision-making and how they had to abide by the community health care system's immense bureaucracy. They also experienced a lack of understanding because the health care services did not have insight or were not interested in the issues that were important to the families.

In the daily struggle against the system, conflicts often occurred with the community health care administrators and the HCP in the home. Many of the families consulted attorneys to obtain the rights they thought they were entitled to. As a result of all of the negative experiences the families had with the system, the participants described how life would be easier for themselves and the ventilator-dependent family member if it was not for all of the problems they had with the community health care services.

#### Quotation examples "Fighting the system" (Core category)

• *"The technical equipment is what scares us the least. It's the human aspect that scares us most. The fight against the system is scarier than if the technical equipment works. Anyone can read the user manual to see how it works." (Daughter, 9)*

• *"There are so many laws, rules, and amendments; it's just horrible. They don't even care about the client. They just run right over them. They sit up in their offices and decide how much help each individual will receive. I just get so angry." (Mother, 5)*

• *"You meet the most arrogant people. You have to convince them that you actually deserve it. Then you're back deciding whether you are doing the right thing. Why are we doing this?" (Father, 6)*

• *"Having a ventilator-dependent child is not so bad, but fighting the community health care services has been much worse. They are the ones that have exhausted me, not having sick children. It's all the bureaucracy. The ones that have been given the diagnosis, they don't know what they are up against. They would have had a good life if it wasn't for the community health care system. It's the community system that has ruined it for us. They are the ones that have set the limitations. It's always the community system." (Mother, 5)*

### Lack of competence and continuity

Ensuring the necessary competence was a big challenge. All of the participants emphasised how much was at risk. One of their loved ones was dependent on life-prolonging ventilator support and, without it, they could die immediately. As a result of this, there was no room for errors while monitoring or during the many complex technical procedures. These procedures must be carried out precisely, because it was important for the families' and the patients' feeling of safety. In addition to this, the main goal was to avoid medical complications, especially airway infections, which could result in hospital admission. Consequently, the families had to ensure that the caregivers were knowledgeable and competent, meaning that those who were suitable for the job had a good attitude and the necessary technical skills.

A common issue for all the families was a lack of continuity in care. Many of the HCP received training, but quit shortly afterwards for various reasons. As a result of this, the families used much of their time training newly employed HCP.

Having an inexperienced, and often unlicensed, HCP had consequences on whether the family members felt safe enough to travel or even leave the house. If HCP called in sick, or if the community health care did not have adequate HCP staffing, family members often had to step in and take shifts.

Owing to the lack of competence and continuity in care, many of the participants believed the community health care services should employ family members as part of the team of carers, but this was usually rejected by the community without substantial explanation. To be able to stay on in one's own home was important for all of the participants, and this was something the families had to fight for because the community health care services believed the patient should live in, or accept help from, public assisted-living facilities or nursing homes.

#### Quotation examples "Lack of competence and continuity"

• *"There is a lot of incompetence. Where are they supposed to get this competence? There is no training for this. You can't learn this anywhere. You can't find training or education to work in a home with such a difficult and demanding job." (Wife, 8)*

• *"I get the impression that everyone that works with him wishes him well. Nice people. But it's more about their attitudes. The people working with this should be of the better kind." (Father, 6)*

• *"I had to constantly share my knowledge and be a mentor to those working with her. And there were a lot of people. Constant turnover for various reasons. And we're not talking about one week of training. We're talking months of training for many of them." (Husband, 10)*

• *"But then there were too little staff to make it work. But that's what happens when they don't hire more people in the community health care services. But then they got hold of some students and we were very pleased with that. We thought it would be great. But until now, we've had 52 caregivers in two years." (Wife, 9)*

### Being indispensable

In addition to the role of being a loving mother, father, wife or daughter, the participants described how they were indispensable to their ventilator-dependent loved ones. This indispensability was described in two ways. Firstly, the families had an intense feeling of responsibility and moral obligation to be there and fight for their loved ones. Their loved ones were vulnerable and helpless individuals because of their huge disabilities and were incapable of taking care of basic needs or demand certain rights. Secondly, the family members' expertise, with regards to their knowledge of medical routines and procedures, was essential for the ventilator-dependent individual's physical and psychological well being and to ensure the necessary quality of care.

The participants described how this indispensible role developed as a result of inadequate support from the community health care services. Due to this, the majority of the participants felt that the care, life and well-being of the ventilator-dependent individual would not be possible without the families' engagement and knowledge.

Families had to be available 24 hours a day. Many of them have not had a holiday since the treatment started. The immense attention they had to give their ventilator-dependent family member took its toll on time spent with other family members. Three of the families with ventilator-dependent children spoke specifically about this because these parents had other children as well. Going on a holiday together was something they wanted more than anything. Yet, the parents expressed how the other siblings were doing well because they made sure to give them plenty of attention.

#### Quotation examples "Being indispensable"

• *"As long as she (her daughter) is fighting, why would we give up? We are able to breath, we are able to eat. But she isn't able to do any of that. She is dependent on machines and human help for food and breathing and getting washed and dressed. As her closest family, it's our duty." (Mother, 2)*

• *"If it wasn't for the knowledge that I have, she would have refused to come home. Of course, after so many years, you learn and adapt to the trials and errors. Her safety is what I know. There's no doubt about that. Everyone knows that, but no one says it. If I had a job then I wouldn't have the opportunity to follow up (in her care). It would have been the end for her then." (Husband, 10)*

• *"If you are sick, you have to have a strong family or else you will succumb. You actually have to be healthy to be sick. The boys have had enough with their illness. And everything else in addition. If it wasn't for me, they would have given up, and they have admitted this themselves." (Mother, 5)*

### Worth fighting for

Despite the daily, and often overwhelming, struggles to ensure their loved ones lived a safe and happy life, the meetings with the families still had a positive undertone. The technology and the families' engagement improved the quality of life for the ventilator-dependent individuals and made it possible for them to be together with the people they loved the most. Many of the participants often compared their own lives with the lives of other "normal" people. They admitted that they lived a very different life, which was now governed by the clock and when certain medical procedures were to be done. But this did not necessarily mean they lived an unhappy life. Despite these hard times, what they had achieved had made them stronger and prouder. Even when stuck in this situation, and many times having to sacrifice their own needs, the family members were able to find a lifestyle that suited all of the limitations in their daily lives. The families had, in a way, become experts in focusing on life's opportunities.

#### Quotation examples "Worth fighting for"

• *"Everyone has problems in their lives that they have to fight through. And you get a little stronger every time. No one lives a completely uncomplicated life. What's most important is to make the best of what you have. Don't regret anything. You could look back in anyone's life and say that things could have been different. But that doesn't help much. We wouldn't have done anything different. I think I have learned so much that I otherwise wouldn't have learned. And we have been able to stay together. That is what we wanted." (Wife, 8)*

• *"Being the caregiver, the nurse, I don't have any problems with that. I think it's nice being together with him. It's a very good experience when I'm together with him." (Father, 6)*

• *"We get up in the morning and drink coffee and talk and read the paper. We'll talk again, and then I'll go do something. Then I do the suctioning of his airways now and then. Maybe we'll go for a walk. We enjoy each other's company. I don't know what we would have done if he were healthy. We've been doing this for so many years. I think it's worth the effort." (Wife, 3)*

## Discussion

This study has revealed that family members have both negative and positive experiences when caring for a ventilator-dependent family member with complex needs at home. Other studies have described comparable experiences [6-8].

Previous research has also illustrated families' problems related to their encounters with the health care system when a family member has complex health care needs [7,17]. Similar to the findings in our study, other studies have described the lack of understanding for the needs of the family [6,18] and that families must often deal with a bureaucratic health care system [19-21]. In the present study, the fight against the system, or the community health care system, was the families' main concern.

This may come as a surprise because Norway is one of the world's richest countries in per capita terms and has a well-developed publicly financed health care system in addition to its strong patients' rights legislation. However, care for these individuals represents enormous challenges for the health care services [22]. This is also confirmed in a recent study where we explored the challenges of the community health services related to advanced HMV in the same communities as the participants in the present study live (manuscript in submission).

In this study, we found that the main causes to the wide range of immense challenges for the community health services seem to correspond with the fact that the patient lives and receives care in his or her private home. Cooperation with family was often very difficult too. Based on our findings, it is apparent that a large gap exists between family members' expectations of what the community health care service is responsible for and what it is actually able to provide. This paradigm could be the result of the community health services' insufficient development and adaptation to technological advancements that make it possible to transfer individuals with complex and intensive care needs out of the hospital, similar to the research findings of the situation in the United Kingdom [17].

Strengthened patient's rights may give rise to moral distress if physicians feel pressured to choose an alternative with which they are not professionally comfortable or which may require seemingly unnecessary resources [23]. It may also be relevant to define whether hospital care at home exceeds the limitations of some of the weakest and most technological-dependent individuals. In the future, the gap between what the health care can potentially do, with unlimited resources, and what they can provide in real life, will rapidly increase [24]. Therefore, in an attempt to direct the resources where they can best contribute to the health and quality of care in Norway, the Norwegian Director of Health [25] has recently signalled a possible reduction and limitation in the provision of extremely costly HMV. No literature was found to support that this is a prioritisation other countries are also considering. If this proposal is implemented in practice, it will most likely create ethical debates.

This study revealed that families play a vital role in ensuring not only the survival, but also the quality of life, for their loved ones. Family members' technological expertise and in-depth knowledge of the ventilator-dependent individual's care needs are essential to guarantee the necessary competence. Similar to other research [6,26], the families in this study criticised the competence of HCP, saying it was insufficient to ensure adequate care. To compensate for this lack of competence, the families kept themselves readily available, which increased their already heavy burdens. As a result of this, the families in this study considered themselves indispensable, similar to what other studies have termed, "being the lifeline" [6].

As long as the families are not offered the necessary support, the question of whether the burdens on families may be too considerable should be asked [8]. The families believe that they have no other choice [6,7], and parallel to this study, the families also felt a moral obligation to be there for their loved ones until the bitter end. The enrichment of being with their loved ones counteracts the enormous responsibility and sacrifices made by the families [7,12] because life is the last thing to give up on [8]. The participants in our study had extensive experience of being the primary caregivers and what kept them going was the strong belief that it was worth it.

Until now, most studies investigated the experiences of families with ventilator-dependent children. This study revealed no major differences in the families' experiences that were dependent on whether the ventilator-dependent individual was a child or an adult. A possible explanation for this is that families of both children and adults share the same dedication and duty for their loved ones because of their strong bonds. In addition, ventilator-dependent children and adults have much in common in relation to their care needs and monitoring. In addition, the complexity of the technical equipment and medical procedures are very similar.

The findings in this study demonstrate the need for measures to support and ease the burden of these families. Patients and families need to be informed of what is to be expected even as early as the initial stages of HMV planning at the hospital [7]. The burdens of HMV care are often underestimated [27]. It is also difficult for families to fully understand the consequences of such a huge lifestyle adjustment before they have experienced it. Even though it is a difficult task, HCP must consider whether the family has the necessary motivation, optimism, and psychosocial resources needed for this type of care [27]. This information would be the central basis for deciding the best location of care for both the patient and the family. The families need to be offered more support from knowledgeable and available HCP [6] in addition to respite services inside and outside the home [7,26].

The participants in the present study had been offered respite services. However, they often rejected this because they did not trust that their loved one would receive the necessary care and monitoring without them present, especially if the respite service was outside of the home. The HCP must also offer the families emotional [5] and psychosocial support [8], like helping the family express their feelings and worries, as well as validating the families' expertise [6,28].

### Study limitations

The sample in this study was small, as is the case with most qualitative studies. In spite of this, we believe this study draws an accurate picture of the current situation in Norway. One must take into consideration that the results of this study may not be transferable to other countries because of the differences in treatment, delegation of responsibilities between families and HCP, organisation and financing of HMV.

## Conclusions

This study has revealed both the negative and positive experiences of families caring for a ventilator-dependent family member with complex care needs at home. This study revealed no major differences in the families' experiences that were dependent on whether the ventilator-dependent individual was a child or an adult. The main finding of this study was the families' strong dissatisfaction with the lack of support and understanding from the community health care services. The findings of this study should remind HCP to validate the families' vital role and accept that their tireless efforts are driven by the sincere wish that their loved ones have the opportunity to enjoy a meaningful life.

The community health care services must act as partners, not as rivals, and meet the family with respect, instead of arrogance. Better continuity, competent personnel and offering respite services are important steps in helping the family to feel less indispensable. This study shows that a large gap exists between the patient's rights and the family's expectations, compared with what is seemingly possible to provide even for a modern health care system in a prosperous country. Expectations from the population are expected to increase. A proposed reduction and limitation in the extremely costly provision of home mechanical ventilation in Norway will trigger new ethical dilemmas. It will, therefore, be necessary to study the ethical dilemmas that these priorities may generate.

## Competing interests

The authors declare that they have no competing interests.

## Authors' contributions

KD, BSB and EWN were responsible for the study conception and design. KD and BSB performed the data collection. KD performed the data analysis and drafted the manuscript. BSB, TT and EWN made critical revisions to the paper for important intellectual content and supervised the study. All authors read and approved the final manuscript.

## Pre-publication history

The pre-publication history for this paper can be accessed here:

http://www.biomedcentral.com/1472-6963/11/156/prepub

## Supplementary Material

Additional file 1 **"Interview guide used for the first in-depth interviews"**. Microsoft Word file (.doc). Click here for file

## References

[B1] Dybwik K Tollåli T Nielsen EW Brinchmann BS Why does the provision of home mechanical ventilation vary so widely? Chron Respir Dis 2010 7 67 73 10.1177/1479972309357497 20015913 PMC2843903

[B2] Dybwik K Nielsen EW Brinchmann BS Home mechanical ventilation and specialized health care in the community: Between a rock and a hard place BMC Health Serv Res 2011 11 115 10.1186/1472-6963-11-115 21605365 PMC3121583

[B3] Kirk S Caring for children with specialized health care needs in the community: the challenges for primary care Health Soc Care Community 1999 7 350 357 11560651 10.1046/j.1365-2524.1999.00197.x

[B4] Marchese S Lo CD Lo CA Outcome and attitudes toward home tracheostomy ventilation of consecutive patients: a 10-year experience Respir Med 2008 102 430 436 10.1016/j.rmed.2007.10.006 18023334

[B5] Kirk S Glendinning C Callery P Parent or nurse? The experience of being the parent of a technology-dependent child J Adv Nurs 2005 51 456 464 10.1111/j.1365-2648.2005.03522.x 16098162

[B6] Mah JK Thannhauser JE McNeil DA Dewey D Being the lifeline: The parent experience of caring for a child with neuromuscular disease on home mechanical ventilation Neuromuscul Disord 2008 18 983 988 10.1016/j.nmd.2008.09.001 18974004

[B7] Carnevale F Alexander E Davis M Rennick J Troini R Daily Living With Distress and Enrichment: The Moral Experience of Families With Ventilator-Assisted Children at Home Pediatrics 2006 117 e48 e60 10.1542/peds.2005-0789 16396848

[B8] van Kesteren RG Velthuis B van Leyden LW Psychosocial problems arising from home ventilation Am J Phys Med Rehabil 2001 80 439 446 10.1097/00002060-200106000-00011 11399005

[B9] Findeis A Larson JL Gallo A Shekleton M Caring for individuals using home ventilators: an appraisal by family caregivers Rehabil Nurs 1994 19 6 11 8159867 10.1002/j.2048-7940.1994.tb01295.x

[B10] Wang KW Barnard A Caregivers' experiences at home with a ventilator-dependent child Qual Health Res 2008 18 501 508 10.1177/1049732307306185 18354049

[B11] Smith SJ Promoting family adaptation to the at-home care of the technology-dependent child Issues Compr Pediatr Nurs 1991 14 249 258 10.3109/01460869109009042 1842789

[B12] Brinchmann BS When the home becomes a prison: living with a severely disabled child Nurs Ethics 1999 6 137 143 10358529 10.1177/096973309900600206

[B13] Ballangrud R Bogsti WB Johansson IS Clients' experiences of living at home with a mechanical ventilator J Adv Nurs 2009 65 425 434 10.1111/j.1365-2648.2008.04907.x 19191941

[B14] Corbin J Basics of qualitative research. Grounded Theory procedures and techniques 1998 second Thousand Oaks, CA: SAGE

[B15] Glaser BG Theoretical Sensitivity. Advances in the Methodology of Grounded Theory 1978 Mill Valley: The Sosiology Press

[B16] Glaser BG A S The Discovery of grounded theory: theories of qualitative research 1967 Mill Valley: Sociology Press

[B17] Kirk S Glendinning C Developing services to support parents caring for a technology-dependent child at home Child Care Health Dev 2004 30 209 18 discussion 219 10.1111/j.1365-2214.2004.00393.x 15104576

[B18] Boström K Ahlström G Living with a chronic deteriorating disease: the trajectory with muscular dystrophy over ten years Disabil Rehabil 2004 26 1388 1398 10.1080/0963-8280400000898 15742985

[B19] Jeppesen J Green A Steffensen BF Rahbek J The Duchenne muscular dystrophy population in Denmark, 1977-2001: prevalence, incidence and survival in relation to the introduction of ventilator use Neuromuscul Disord 2003 13 804 812 10.1016/S0960-8966(03)00162-7 14678803

[B20] Brinchmann BS "Facing the system": parents of seriously disabled children and the helping agencies Psychology of Coping 2005 New York: Nova Science Publishers 1 16

[B21] Lindahl B Sandman PO Rasmussen BH On being dependent on home mechanical ventilation: depictions of patients' experiences over time Qual Health Res 2006 16 881 901 10.1177/1049732306288578 16894222

[B22] Vitacca M Escarrabill J Galavotti G Vianello A Prats E Scala R Peratoner A Guffanti E Maggi L Barbano L Balbi B Home mechanical ventilation patients: a retrospective survey to identify level of burden in real life Monaldi Arch Chest Dis 2007 67 142 147 18018753 10.4081/monaldi.2007.485

[B23] Førde R Aasland OG Moral distress among Norwegian doctors J Med Ethics 2008 34 521 525 10.1136/jme.2007.021246 18591286

[B24] Guldvog B Strengthening quality of care in four Nordic countries Scand J Public Health 2009 37 111 116 10.1177/1403494809102311 19244391

[B25] The Norwegian Council for Quality Improvement and Priority Setting in Health Care Presentation in English 2008

[B26] Heaton J Noyes J Sloper P Shah R Families' experiences of caring for technology-dependent children: a temporal perspective Health Soc Care Community 2005 13 441 450 10.1111/j.1365-2524.2005.00571.x 16048532

[B27] Glass C Grap MJ Battle G Preparing the patient and family for home mechanical ventilation Medsurg Nurs 1999 8 99 101 104-107 10410007

[B28] Kirk S Glendinning C Supporting 'expert' parents-professional support and families caring for a child with complex health care needs in the community Int J Nurs Stud 2002 39 625 635 10.1016/S0020-7489(01)00069-4 12100874

