# Supplementary material for: "Fighting the system": Families caring for ventilator-dependent children and adults with complex health care needs at home
Source: BMC Health Serv Res. 2011 Jul 4;11:156. doi: 10.1186/1472-6963-11-156 (PMC3146406; doi:10.1186/1472-6963-11-156)
Supplement: Additional file 1 — "Interview guide used for the first in-depth interviews". Microsoft Word file (.doc). [file 1472-6963-11-156-S1.DOC]

**Appendix**

**Interview guide used for the first in-depth interviews:**

1. How does it feel to have a close family member that is sick and dependent on a ventilator to survive?
2. What are the biggest problems/challenges when giving care to a ventilator-dependent family member?
3. Which consequences do the treatment and the major focus of the sick have on your own life?
4. How is the cooperation with community health care services?
5. Is it worth the effort and what are the positive sides of living a life so special?
6. Is there anything else you would like to add that we have not talked about today?
